# Intermittent hypoxia training does not change erythrocyte aggregation indicators in young, healthy men

**DOI:** 10.3389/fphys.2024.1386650

**Published:** 2024-06-25

**Authors:** Mateusz Mardyła, Marcin Maciejczyk, Tomasz Pałka, Magdalena Więcek, Justyna Kusmierczyk, Jadwiga Szymura, Zbigniew Szygula

**Affiliations:** ^1^ Department of Physiology and Biochemistry, University of Physical Education, Kraków, Poland; ^2^ Department of Clinical Rehabilitation, University of Physical Education, Kraków, Poland; ^3^ Department of Nutrition and Sport Medicine, University of Physical Education, Kraków, Poland

**Keywords:** hypoxia, blood rheology, erythrocyte aggregation, interval training, altitude

## Abstract

**Background:**

The increasing popularity of hypoxic training as a training method to improve physical performance indicates the need to study the effects of this type of intervention on blood morphological and rheological indices, since the adaptive changes that follow such training mainly affect blood indices. In this study, the effects of a 4 weeks of intermittent hypoxic training on blood morphological and rheological indicators in physically active men were assessed.

**Methods:**

Forty-eight young, physically active men, participated in the study. Participants were randomly divided into three groups: two training groups and a control group without intervention (CTRL). Each group consisted of 16 participants. Training groups performed interval training (three times per week, 4 weeks, 12 workouts) under different conditions: in hypoxia (IHT; fraction of inspired oxygen (FiO_2_) = 14.4%) or in normoxia (NT; FiO_2_ = 20.9%). The control group performed only two workouts 4 weeks apart. Blood was taken during the first and last training session at rest, and 3 minutes after training.

**Results:**

After the last training session, there was a significant increase in mean corpuscular volume and a decrease in mean corpuscular haemoglobin concentration measured at rest only in the IHT group. There was also a significant decrease in resting aggregation amplitude for the IHT and CTRL groups. There was no difference in change of post-exercise plasma volume between first and last training session.

**Conclusion:**

The applied intermittent interval training in conditions of normoxia and hypoxia had no significant impact on resting aggregation parameters. This suggest that training under hypoxic conditions does not cause adverse rheological changes.

## 1 Introduction

To achieve success in many sport disciplines, there is a necessity to apply the most effective methods of training together with proper recovery time, diet regime and sufficient sleep time. One of this method is the Live Low-Train High (LL-TH) model – related to training in environmental conditions with oxygen deficiency. This training model has much greater application due to easier introduction into an athlete’s standard training plan (Wilber, 2001). There are several models of artificial simulated hypoxic training were proposed in the past, that could potentially increase athletes’ performance (Wilber, 2001; McLean et al., 2014). Nevertheless, some of these models characterise greater effectiveness than others. In one of the most recent meta-analyses, it was shown that the generally most applied method in the population is intermittent hypoxia training (IHT) (Yu et al., 2023).

The main effects of hypoxia training are due to improvement in muscle tissue efficiency, resulting in increased aerobic and anaerobic performance (Zoll et al., 2006; Hamlin et al., 2018; Ambrozy et al., 2020). Adaptations to hypoxia are achieved by the activation of Hypoxia Inducible Factors (HIF), especially HIF-1. HIF1-α accumulation in various cells results in increased erythropoiesis, angiogenesis and mitochondrial biogenesis (Semenza et al., 2006). Thus, hypoxia initiates erythropoietin (EPO) gene transcription (Semenza, 2004), however, the quantitative effect of red blood cell (RBC) production is linked with duration of hypoxia exposure. An increase in red cell mass is usually observed in the Live High-Train Low or Live High-Train High methods (Clark et al., 2009), however increase of reticulocytes as early marker of erythropoiesis can be seen within 1–2 days after endurance training even in normoxia condition (Schmidt et al., 1988). Erythropoietin action besides of stimulating erythropoiesis consist in binding to EPO receptors (EPO-R) located on human erythroid colony - forming cells thus stimulating proliferation and prevents from apoptosis of newly formed cells (Sui et al., 2000; Günter et al., 2013). Regular training lead to increase of mRNA expression for erythropoietin and for EPO receptor in bone marrow (Suzuki, 2015). Despite this, there is no much data regarding the impact of prolonged hypoxia training on red blood cell hemorheology (Lin et al., 2021; Park et al., 2022a; Teległów et al., 2022). Exercise in hypoxia at different oxygen condition did not altered red blood cell aggregation in healthy male despite of significant increase blood lactate concentration (Moon et al., 2016). The ability of lactate to increase mitochondrial biogenesis has been previously described (Pellerin et al., 2022). Hypoxia induces diverse adaptive mechanisms, and those most commonly studied involve erythropoietin, mitochondrial biogenesis or HIF. Such studies related to hemorheology have not been carried out in people who are active physically but do not practice sports competitively.

Hemorheology is related to mechanical and biochemical properties of red blood cells circulating in a human’s bloodstream. RBC deformability, together with aggregation and blood plasma viscosity, can determine oxygen delivery especially to the muscles during exercise (Brun et al., 2010; Connes et al., 2013). RBC and fibrinogen aggregation comprise an important component of blood viscosity. Erythrocyte aggregation can influence haemodynamics, RBC distribution and flow dynamics in the microcirculation level, particularly at low shear stress. The higher the degree of aggregation, the lower the tissue perfusion and aerobic capacity (Brun et al., 2010). In general, RBC shows low or moderate tendencies towards aggregation in healthy people (Moon et al., 2016). However, exposure to intermittent hypoxia training can also lead to impairment in blood rheology by increasing rigidity of erythrocytes or modulation in ion transport channel activity (Mao et al., 2011; Grygorczyk and Orlov, 2017). Moreover, some hemorheology indices, such as deformability, can vary depending on the group of participants, mainly with regard to their level of physical activity. As shown in *in vitro* examinations, lactate decreases red cell deformability in non-trained sedentary participants, but surprisingly, its level increases in well-trained individuals. This fact could be explained as more effective lactate transport by monocarboxylate transporters (Connes et al., 2004).

There is not much knowledge concerning the impact of intermittent hypoxic training on the rheological properties of erythrocytes. Based on previously published reports, we hypothesized that intermittent hypoxia training leads to greater improvement in haemorheological variables than training in normoxia (decrease in aggregation index, increase in half-life kinetics of aggregation time). Therefore, this study was aimed to evaluate the effects of intermittent training in hypoxia on haemorheological and morphological variables in young, physically active men.

## 2 Materials and methods

### 2.1 Study design

The sample size was determined *a priori* using G*Power version 3.1.9.7 (Dusseldorf, Germany). The following options were selected in the software: test family = f tests; statistical test = ANOVA-repeated measures, within–between interactions; type of power analysis = computed required sample size—given α, power and effect size. Input parameters into the software were as follows: effect size f: 0.25; α error probability: 0.05; power: 0.85; number of groups: 3; number of measurements: 2; correlation among measures: 0.5; nonsphericity correction: 1.0. The required sample size was 16 participants per group (total sample size = 48). Participants were randomly divided into three groups: two performing the same interval training (three times a week for 4 weeks): in normoxia (NT) (200 m above sea level, fraction of inspired oxygen (FiO_2_) = 20.9%) and in normobaric hypoxia (IHT) conditions (FiO_2_ = 14.4%); and the control (CTRL). Each group consisted of 16 participants. In total, the IHT and NT groups performed 12 training sessions while the CTRL group performed only two training bouts (the first and last training, 4 weeks apart to keep the same time). Blood samples were taken before and after the first and final training session.

To determine individual training workloads, the participants performed in normoxia an incremental test on a cycle ergometer until volitional exhaustion (GE, eBike, Germany). In the graded test, the first ventilatory threshold (VT1), second ventilatory threshold (VT2), were determined using a metabolic cart (Metalyzer, Cortex, Germany), as previously described (Maciejczyk et al., 2023). On the basis of the results of the graded test, the training loads corresponding to absolute power output at VT1 and VT2 were established individually (Maciejczyk et al., 2023).

Somatic measurements were performed on the same day as the graded test. These measurements included body height (BH), body mass (BM) and percentage of body fat (%BF). Body height was estimated using stadiometer (Seca, Germany). Body mass and body composition were assessed using a body composition analyser (IOI-353, Jawon, Korea).

The study was approved by the Bioethics Commission of the Regional Medical Chamber in Kraków, Poland (opinion No. 47/KB/OIL/2022), and it was carried out in accordance with the 1964 Declaration of Helsinki. All participants provided written, informed consent after being familiarized with the study protocol. Participants could withdraw from the project at any stage.

### 2.2 Participants

Forty-eight healthy, young men were recruited for the research experiment (16 participants per group). Before proceeding, the participants underwent initial medical examination (e.g., electrocardiogram at rest and during exercise, complete blood count, haemoglobin and iron status). The qualified participants had not participated in competitive sports, in other forms of physical training and had not been subjected to hypoxia exposure within the 6 months prior to the experiment. Their physical activity was varied and spontaneous, but not exceeding three times a week. The participants were required not to change their diet or physical activity during the study. They were between the age of 19 and 26, and the groups did not differ (*p* > 0.05) in body mass or body composition. Mean body height was measured in each group (CTRL, NT, IHT): 178.9 ± 5.9, 179.7 ± 5.6 and 182.0 ± 5.5 cm; as well as body mass: 75.8 ± 11.5, 76.7 ± 8.3 and 79.6 ± 10.4 kg; %BF 18.3 ± 5.3, 17.6% ± 3.9% and 17.4% ± 3.9%, respectively.

### 2.3 Training

Interval training was performed for 4 weeks on Mondays, Wednesdays and Fridays (12 workouts) in a hypoxic thermoclimatic chamber (Hypoxico, Germany) or in conditions of normoxia, on bicycle ergometers (Wattbike, United Kingdom) and lasted 60 min. The training consisted of a warm-up (6 min) with a power output at VT1 followed by six series of efforts lasting 6 minutes with an active recovery duration of 3 minutes (2:1 ratio). The work load of the efforts was set individually and corresponded to the power output at VT2 (effort) and VT1 (active recovery). All training sessions took place in the same temperature of 21 ± 0.5°C and humidity of 40% ± 1%. During the intervention, the load (power) was not corrected.

### 2.4 Blood sampling and blood analysis

Blood samples was taken four times overall (for every group):

- Twice at the 1^st^ training session: at rest, about - 15 min before the beginning of training (T1) and 3 minutes after training had finished (T2);
- Twice on the last, 12th training session: at rest about 15 min before the beginning of training (T3) and 3 minutes after training had finished (T4).

The CTRL group underwent only two training sessions: the first (at the beginning) and second (at the end of the experiment), but the time sequences of blood sampling were the same as in IHT and NT groups.

The blood samples were collected from the ulnar vein by a nurse, in the total amount of approximately 5 ml. The blood morphology indicators including red blood cell count (RBC), haemoglobin concentration (HGB), haematocrit (HCT), mean corpuscular volume (MVC), mean corpuscular haemoglobin (MCH), mean corpuscular haemoglobin concentration (MCHC), reticulocytes (RET) [‰ and mln/mm^3^] were obtained using fluorescence flow cytometry with the Sysmex XN10 (for red blood cells and dependent variables, e.g., Hgb) and Sysmex XN (for reticulocytes) haematology analysers (Sysmex Corporation, Japan). The aggregation parameters (aggregation index (AI), amplitude of aggregation (AMP) and half-time kinetics of total aggregation (T1/2) were tested with the Lorrca Maxsis Osmoscan (Lorrca^®^, RR Mechatronics, AN Zwaag, Netherlands) and according to the method proposed by Hardeman et al. (2001). Plasma volume change (%PV), as a post-exercise effect, was calculated using the formula by Dill and Costill (1974), further modified by Harrison et al. (1982).

### 2.5 Statistical analysis

Data are presented as mean and standard deviation or mean and standard error of measurement. Data distribution was checked using the Shapiro–Wilk test. Homogeneity of variance within the groups was tested via Levene’s test. ANOVA with repeated measures was used to assess the effects of training, effect size and inter-group difference. If a significant (*p* < 0.05) effect of the main factor (group, time or interaction group-time) was found in the ANOVA, *post hoc* analysis was performed using the Tukey test. The STATISTICA 13.3 package (StatSoft, Inc., Tulsa, OK, United States) was used for calculations.

## 3 Results

### 3.1 Selected blood morphology parameters

There was a significant effect of intervention (time) in the MCV (*p* < 0.001) and MCHC (*p* < 0.001) variables. Moreover, further analysis demonstrated an interaction between time x group for the MCV (*p* < 0.001) and MCHC indices (*p* < 0.001). Post-hoc analysis showed a significant increase in MCV and a decrease in MCHC, but only in the IHT group. For the other studied variables, no significant changes were recorded over time (Table 1).

**TABLE 1:** Selected blood morphology indicators noted in participants for first and last training sessions.

Variable	Group	Rest, first training (T1)	Rest, final training (T3)	Effect: group F p	Effect: time F p	Interaction F p	Change Pre vs. Post: post hoc p
RBC [mln/mm^3^]	IHT	4.94 ± 0.31	4.88 ± 0.37	2.87 0.07	6.97 0.01	0.29 0.75	NS
	NT	5.17 ± 0.38	5.06 ± 0.34				NS
	CTRL	5.21 ± 0.39	5.15 ± 0.31				NS
HGB [g/dl]	IHT	15.09 ± 0.90	14.89 ± 1.04	2.44 0.10	4.87 0.03	0.44 0.65	NS
	NT	15.83 ± 0.92	15.52 ± 0.86				NS
	CTRL	15.58 ± 1.12	15.48 ± 0.96				NS
HCT [%]	IHT	44.03 ± 2.49	44.58 ± 3.02	1.98 0.15	0.58 0.45	2.12 0.13	-
	NT	46.16 ± 2.89	45.53 ± 2.58				-
	CTRL	46.14 ± 2.87	45.63 ± 2.33				-
MCV [fl]	IHT	89.29 ± 3.49	91.48 ± 3.13	1.67 0.20	37.07 <0.001	16.15 <0.001	<0.001
	NT	89.43 ± 2.26	90.09 ± 2.44				NS
	CTRL	88.6 ± 2.86	88.65 ± 2.52				NS
MCH [pg]	IHT	30.6 ± 1.44	30.58 ± 1.33	1.90 0.16	1.24 0.27	1.20 0.31	-
	NT	30.68 ± 0.96	30.71 ± 0.91				-
	CTRL	29.98 ± 1.02	30.06 ± 1.08				-
MCHC [g/dl]	IHT	34.28 ± 0.52	33.41 ± 0.78	1.30 0.28	16.35 <0.001	15.62 <0.001	<0.001
	NT	34.33 ± 0.79	34.09 ± 0.90				NS
	CTRL	33.74 ± 0.85	33.92 ± 0.92				NS
RET [‰]	IHT	12.46 ± 2.69	12.84 ± 2.84	1.25 0.30	0.16 0.69	0.65 0.53	-
	NT	14.44 ± 4.40	13.85 ± 3.18				-
	CTRL	14.42 ± 3.38	14.19 ± 4.06				-
RET [mln/mm3]	IHT	0.061 ± 0.014	0.064 ± 0.015	1.92 0.16	0.43 0.52	1.10 0.34	-
	NT	0.075 ± 0.023	0.071 ± 0.018				-
	CTRL	0.074 ± 0.018	0.073 ± 0.23				-

Data are presented as mean values ± SD. RBC: red blood cells; HGB: haemoglobin; HCT: haematocrit; MCV: mean corpuscular volume; MCH: mean corpuscular haemoglobin; MCHC: mean corpuscular haemoglobin concentration; RET: reticulocytes; NS, not significant vs. 1^st^ training session).

### 3.2 RBC aggregation parameters

**FIGURE 1:**
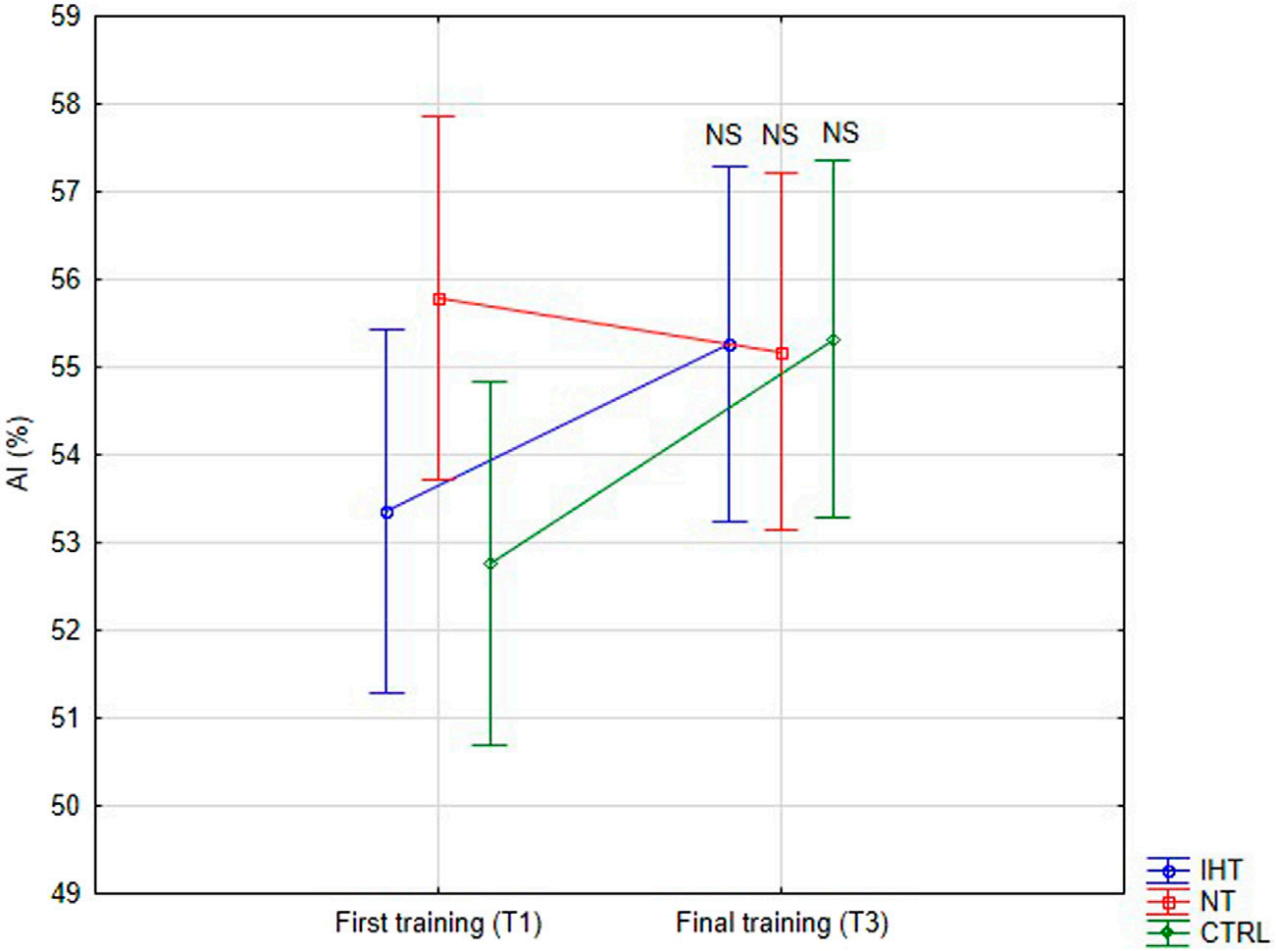
Red blood cell aggregation index (AI) at first and final training sessions during rest (NS - not significant).

**FIGURE 2:**
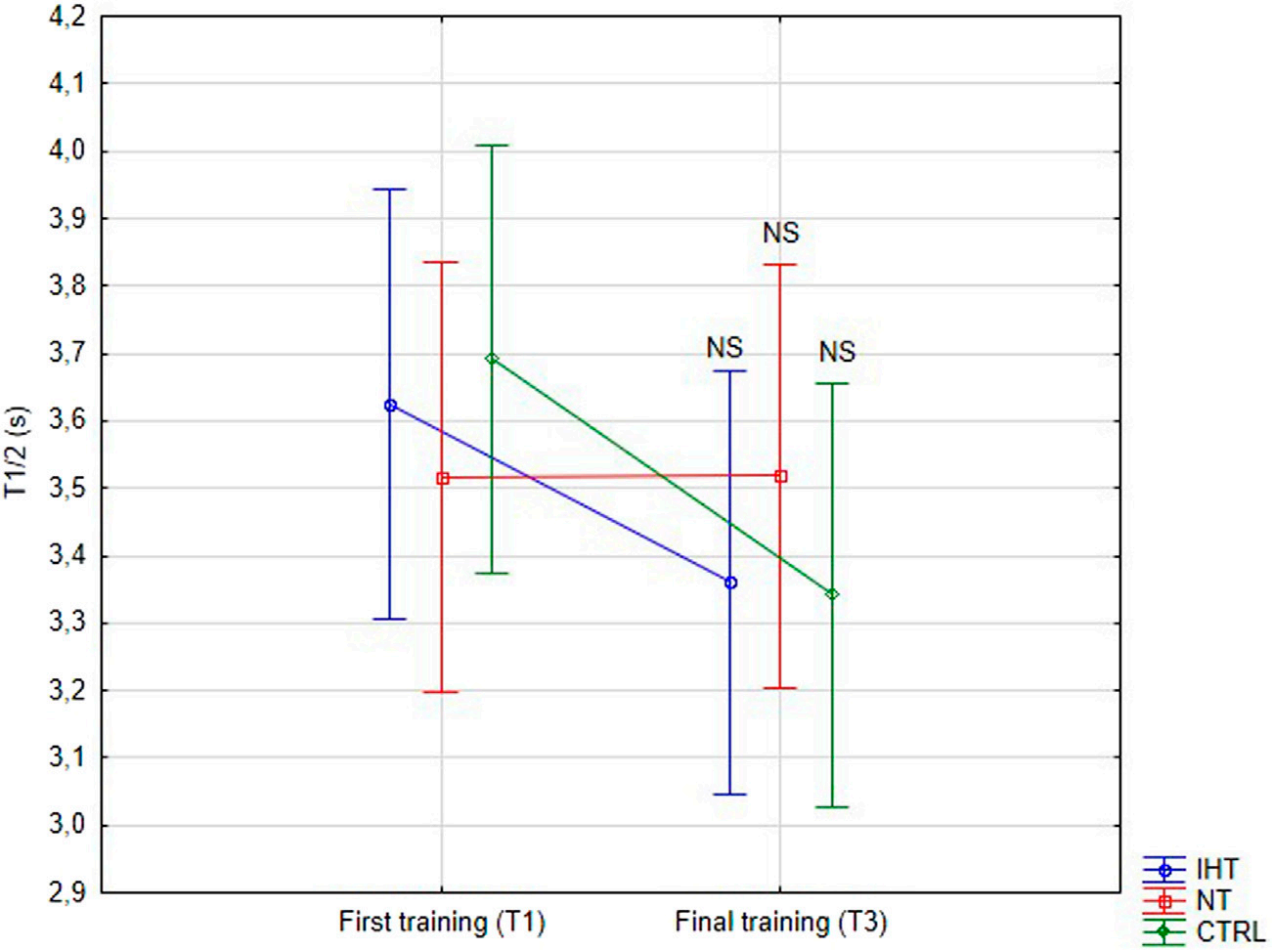
Red blood cell half-life of total aggregation (T1/2) at first and last training sessions during rest (NS - not significant).

**FIGURE 3:**
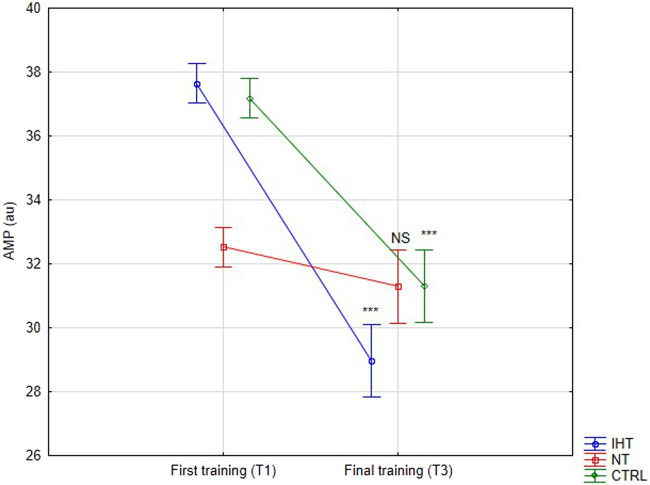
Red blood cell aggregation amplitude (AMP) at first and last training sessions during rest (***p < 0.001, NS- not significant).

There was no significant difference between groups, time or interaction group x time in aggregation index, or in half-life of total aggregation but there was significant interaction group x time in amplitude aggregation. IHT and CTRL group has lower AMP between first and final training (Table 2; Figures 1–3).

**TABLE 2:** Effects of interval training on resting erythrocyte aggregation parameters in three groups measured during first and last training sessions.

Variable	Group	Rest, first training (T1)	Rest, final training (T3)	Effect: group F p	Effect: time F p	Interaction F p	Change Pre vs. Post: post hoc p
AI [%]	IHT	53.37 ± 6.51	55.26 ± 7.58	0.16 0.85	2.20 0.15	1.26 0.29	-
	NT	55.79 ± 11.23	55.18 ± 9.94				-
	CTRL	52.77 ± 6.08	55.32 ± 6.47				-
T1/2 [s]	IHT	3.63 ± 0.96	3.36 ± 1.14	0.002 0.99	2.68 0.11	0.72 0.49	-
	NT	3.52 ± 1.78	3.52 ± 1.61				-
	CTRL	3.69 ± 0.88	3.34 ± 0.92				-
AMP [au]	IHT	37.64 ± 1.64	28.97 ± 5.50	2.67 0.09	64.16 <0.001	10.87 <0.001	<0.001
	NT	32.53 ± 2.90	31.29 ± 2.80				NS
	CTRL	37.17 ± 2.69	31.30 ± 4.90				<0.001

Data are presented as mean values ± SD. AI: aggregation index; T1/2: half-life kinetics of aggregation; AMP- amplitude aggregation; NS, not significant vs. 1^st^ training session).

There was no significant change in exercise diffrence regarding aggregation variables during the 1st and 12th training sessions (Table 3). The effect of single exercise on aggregation parameters did not differ between first and last training sessions in any of the experimental groups.

**TABLE 3:** Exercise diffrences in red cell aggregation variables between the first and final training sessions.

Variable	Group	∆(Ex-Rest) First training	∆(Ex-Rest) Final training	Effect: group F p	Effect: time F p	Interaction F p	Change 1st vs. 12th training *post hoc* p
AI [%]	IHT	3.53 ± 3.36	3.67 ± 6.98	1.78 0.18	0.09 0.76	0.59 0.56	-
	NT	2.79 ± 2.74	1.85 ± 4.57				-
	CTRL	0.48 ± 5.35	2.21 ± 5.05				-
T1/2 [s]	IHT	−0.50 ± 0.48	−0.48 ± 0.99	1.12 0.34	0.03 0.87	0.72 0.49	-
	NT	−0.47 ± 0.45	−0.29 ± 0.84				-
	CTRL	−0.05 ± 1.00	−0.32 ± 0.76				-
AMP [au]	IHT	0.44 ± 1.33	−0.51 ± 6.89	0.05 0.94	0.18 0.67	1.18 0.32	-
	NT	−0.69 ± 3.96	1.32 ± 2.11				-
	CTRL	0.21 ± 2.30	0.18 ± 5.38				-

Data presented as mean values ± SD., Ex: after training; Rest: before training; AI: aggregation index; T1/2: half-life kinetics of aggregation, AMP-amplitude aggregation.

### 3.3 Plasma volume change

**FIGURE 4:**
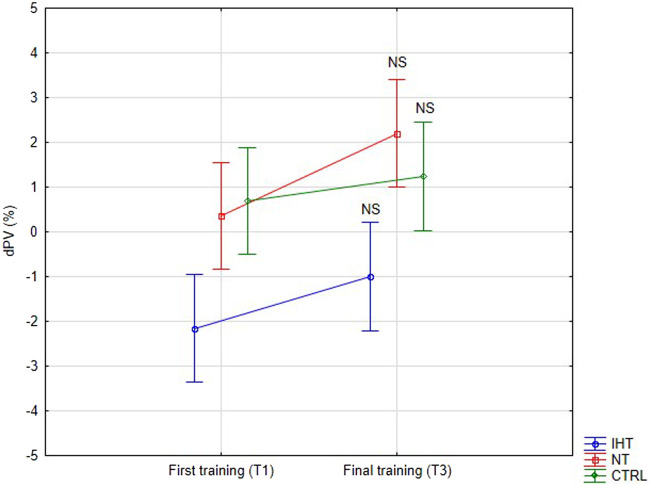
Percentage changes in plasma volume on first and final training session as result of exercise (NS - not significant).

There were no significant changes or interactions between the first and final training sessions, however, after training in normoxia (CTRL and NT groups), an increase was noted in plasma volume (PV) compared to the IHT group, for which the decrease in PV was observed after both training sessions (Figure 4; Table 4).

**TABLE 4:** Blood plasma changes as effect of first and final training sessions.

Variable	Group	∆ (Ex-Rest) first training	∆ (Ex-Rest) final training	Effect: group F p	Effect: time F p	Interaction F p	Change Pre vs. Post: post hoc p
Change PV (%)	IHT	−2.15 ± 3.72	−1.00 ± 5.55	2.52 0.09	2.26 0.14	0.23 0.80	-
	NT	0.35 ± 5.11	2.20 ± 4.12				-
	CTRL	0.70 ± 5.30	1.24 ± 4.71				-

Data are presented as mean values ± SD. PV: plasma volume; Ex: after training; Rest: before training.

## 4 Discussion

Modern training models related to hypoxia exposure (such as Living Low-Training High) could affect blood circulation and the haemorheological system. The findings of this study are partly contradictory to those in papers which ascertain a decrease in red cell aggregation as one of the effects of hypoxia training and thereby, improvement in hemorheology (Jung et al., 2020; Park et al., 2022a). This could be explained by the methodological assumptions of this study - blood samples were taken immediately after the last training. Other authors most often analysed these variables a few days after completing the training plan (Teległów et al., 2022), thus, there is more time for some adaptation changes. The current study was designed to assess response to submaximal interval training after IHT in male participants, rather than just resting data measured a few days after training.

There is consensus that the effects of endurance training on haematology indicators in athletes usually lead to a decrease of relative quantitative red blood cell parameters. Although we did not note changes in PV in our study, the most frequent reason for this is post-training enhancement of plasma volume occurring within 3 hours after exercise and persisting a few days (Gillen et al., 1991). The effect of haemodilution is inextricably linked to the phenomenon of sports anaemia, often observed among professional athletes and even Olympians (Clement et al., 1987; Weight et al., 1992).

Our results confirm that IHT does not lead to increased blood cell counts (Karlsen et al., 2002; Abellán et al., 2005; Czuba et al., 2018). For the acceleration of erythropoietin response, it has been proposed that hypoxic doses should be equivalent to exposure on hypoxia for a minimum of 12–16 h/day (Wilber et al., 2007). On the other hand, an increase has been observed in the mean corpuscular volume of red blood cells, but only in the IHT group, which is evidence of augmented, young blood cells in the bloodstream or the adaptation of old ones to release damaged protein molecules in the vesiculation process (Bizjak et al., 2015). This is in line with the results presented by Czuba et al. (2011). These authors noted that mean corpuscular volume was also increased after 3 weeks of IHT in cyclists. However, the remaining haematological variables were not affected. A slight effect of increased erythropoiesis could be observed by a minor increase in reticulocyte count only in the IHT group, in contrast to the other groups.

Acute effects of single exercise bouts in different normobaric hypoxia conditions (various oxygen concentrations) were studied by Moon et al. (2016). None of the investigated hypoxia conditions affected red cell aggregation despite an increase in blood lactate along with lower oxygen concentration. Similar results of aggregation were obtained by Connes et al. (2007), who also did not find any correlations with lactate increase as a result of maximal exercise in trained athletes (the experiment was performed *in vitro*). Moreover, a decrease in deformability was observed, but only in 11.5% subjected to the O_2_ condition, and it was indicated that exposure and exercise on moderate altitude (most often abuse among competitive athletes) do not cause disturbances in hemorheology (Moon et al., 2016). There were also not changes in aggregation after 30 min of submaximal exercise, independently of hypoxic condition (Park et al., 2022b). Partly opposite effects were shown after performing a hypoxic exercise test by Lin et al. (2021). Global RBC aggregation was increased in each group after a cardiopulmonary test in hypoxic conditions (12% O_2_); however, after 6 weeks of training in hypoxia conditions, a marked increase in fibrinogen binding probability was observed, unlike in the case of the same training performed in normoxia. Additionally, resting aggregation values were increased only in the hypoxia training group. The authors explained the fact of exercise aggregation increase as a result of an increase in intrinsic erythrocyte factors Lin et al. (2021). The study performed on small group of eight, well-trained athletes showed that 30-min submaximal exercise at a 10% intensity above VT1 lead to disruption in hemorheology, decreasing red cell deformability and increasing aggregation (Nemkov et al., 2021). In contrast, there was no control group in this study, therefore, the results cannot be fully comparable. The result of the aggregation index obtained after completing the 1st and 12th training sessions in the present study did not differ from resting values, however, there was a non-significant, small increase in each group after exercise, with the largest difference noted in the IHT group. This fact can be partly explained by the potentially largest fluid loss and haemoconcentration phenomenon that occurred in hypoxia group but was not observed in the normoxia and control groups. This may confirm that relative intensities were higher in the IHT group, causing haemoconcentration and the greatest increase in aggregation index.

The effects of training in hypoxia on erythrocyte aggregation are ambiguous. In the current trial, no significant changes in resting aggregation parameters were ascertained, although the aggregation index (AI) was increased during the final training session in the IHT and CTRL groups compared to the NT group, for which this value was minimally lower in relation to the first training bout. However, we find decrease of AMP parameter in IHT and CTRL group which indicate slight hemorheology alteration, potentially related to body hydration status. Similarly, Teległów et al., 2022 found that 3-week, intermittent hypoxia training did not influence aggregation index or half-life of aggregation in young professional rowers. However, the amplitude of aggregation (AMP) was increased during the 2^nd^ second examination in the group that performed training in normoxia conditions. Moreover, red cell deformability was heightened in both groups, indicating no extra/additional effect of hypoxia on the mechanical properties of erythrocytes. In the paper by Park et al., 2022a, the authors described interval training performed among women and it was shown that both forms (training in normoxia and hypoxia) resulted in improvement regarding aggregation and deformability. However, a greater effect was observed in the hypoxia group. Similar results were obtained by Jung et al. (2020) for a 12-week training plan under conditions of hypoxia. These findings cannot be confirmed in the present study as the experiment was performed in men, over a shorter period of time (4 weeks), and normobaric hypoxia was used, unlike in previous study, where hypobaric hypoxia was applied (Park et al., 2022a). The authors of the aforementioned publication did not directly explain the mechanism of this hemorheology enhancement. Instead, they indirectly underlined the issues related to improvement of arterial endothelial function and metabolic processes (Jung et al., 2020; Park et al., 2022a).

To conclude, despite there only being little data on the red cell aggregation with regard to training in conditions of hypoxia, it is worth noting that these effects could be dependent on gender and health status. The presented results are closer to those obtained by Lin et al. (2021), who showed an increase in aggregation after IHT in men, whereas the remaining trials carried out among women feature decreases in red blood cell aggregation after the same procedure (Jung et al., 2020; Park et al., 2022a). This potential dependency and unidentified factors which affect these changes require further analysis.

### 4.1 Limitation of the study

In this research, the authors did not obtain red cell deformability results or other haemorheological indicators, such as fibrinogen or plasma viscosity. However, the change in plasma volume was determined. To fully obtain a haemorheological image of ongoing modifications during the experiment, it is necessary to apply the remaining methods, such as deformability and plasma/blood viscosity measurements.

## 5 Conclusion

The 4-week, intermittent, interval training in conditions of normoxia and hypoxia had no significant impact on resting aggregation parameters (AI, T1/2). It can be assumed that 4 weeks of IHT was too short a time and too weak a stimulation to induce significant haemorheology changes, even in previously non-trained, healthy participants.

## Data Availability

The original contributions presented in the study are included in the article/Supplementary Material, further inquiries can be directed to the corresponding author.
